# Lower urinary tract symptoms and erectile dysfunction in men from the Republic of Moldova

**Published:** 2018

**Authors:** I Dumbraveanu, E Ceban, P Banov

**Affiliations:** * Department of Urology and surgical nephrology, State University of Medicine and Pharmacy „Nicolae Testimiţanu” of the Republic of Moldova

**Keywords:** lower urinary tract symptoms, erectile dysfunction, men’s health, correlation

## Abstract

A transversal study based on 1,186 respondents’ group questioning was realized in order to study lower urinary tract symptoms (LUTS) and erectile dysfunction (ED) prevalence in men from the Republic of Moldova. The basic tools used included IPSS (International Prostate Symptom Score) and IIEF-5 (International Index of Erectile Function) questionnaires as well as general questions to assess the presence of risk factors.

ED total prevalence in the studied population was 47.1%, inclusively mild form - 22%, slightly moderate - 12.4%, moderate - 7.6% and severe - 51%.

The overall prevalence of LUTS symptoms, according to the IPSS score, was 42.9% with significant variations in males up to 40 years - 15.7% and over this age - 62.6% (p <0.05). LUTS presence correlates directly with erectile dysfunction prevalence, especially in men over the age of 40, where 82% of those with LUTS will also have ED.

LUTS and ED symptomatologies have an increased prevalence among men from the Republic of Moldova, especially over the age of 40. Statistically significant correlations between these two pathologies were demonstrated. These correlations need to be taken into account both in diagnosis establishment and in concomitant treatment.

## Introduction

Erectile dysfunction (ED) affects about half of men over the age of 50. At the same time, clinical manifestations of urinary tract diseases, such as nocturnal pollakiuria, strangury, dysuria, along with others are manifested more frequently after this age. Epidemiological studies show direct correlations between erectile dysfunction and lower urinary tract symptoms (LUTS), regardless of age or other comorbidities such as diabetes or high blood pressure. However, until now it has not been established with certainty whether erectile dysfunction is a consequence of low urinary symptomatology or, conversely, LUTS symptomatology appears as an erectile dysfunction. The existence of a direct association between erectile dysfunction and LUTS, considering that both have the age as a common point, was confirmed by both epidemiological studies and explanations of pathophysiological mechanisms at the beginning of the 21st century.

The first studies on the correlations between LUTS and erectile dysfunction showed that 72% of men with ED suffer concomitantly from lower urinary symptomatology [**1**][**2**].

The MSAM-7 study (Multinational Survey of Aging Male), which took place in the US and six European countries, included 12,815 patients aged between 50 and 80. About 90% of respondents reported LUTS symptoms, but only 19% have sought medical consultation, and only 11% followed treatment. One of the main findings of the study was that LUTS severity is a major risk factor for sexual dynamic dysfunction disorders affecting primarily the erectile component [**1**][**2**].

Subsequent studies, including large groups, confirmed this interdependence. Wein showed on a group of 2,954 patients with a global prevalence of erectile dysfunction in patients with LUTS of 49%, but LUTS severity was a strong erectile dysfunction predictor of 8.90 (p <0.05) probability ratio. LUTS presence or its change to a higher severity category has a greater impact on erectile function compared with 10 years of aging, but about 24.8% of patients have no sexual activity because of LUTS [**3**].

In 2012, Seftel published a meta-analysis based on 23 relevant scientific studies, which showed that up to that moment there were few population surveys that used IPSS and IIEF questionnaires assessing global prevalence and correlations between diseases. However, based on some alternative evaluations, it was established that among the general male population between 13-29% had moderate to severe LUTS symptomatology, but 8-35% had ED from moderate to severe. The coexistence between LUTS and ED of any severity was 71-80% of men who requested treatment for LUTS. These correlations increase with age ranging from 59-86% in men aged 40-60 years and from 79 to 100% in men over 50 years of age [**4**].

Most studies conducted up to 2000 accentuated a single risk factor for LUTS and erectile dysfunction – the age. The following studies, including recent ones, showed that several risk factors are similar in both situations, especially metabolic syndrome, hormonal disturbances, diabetes and others. It is certain that both situations share at least four pathophysiological mechanisms: change in bioavailability of nitric oxide, alpha adrenergic receptor hyperactivity, pelvic atherosclerosis and sexual hormones disorder [**5**].

Due to the fact that causal mechanisms are common, it was proposed that the men who require consultation for pathology be investigated in conjunction with the mandatory assessment of sexual function. On the other hand, more clinical studies showed LUTS improvement after the administration of phosphodiesterase-5 inhibitors. However, in cases of testosterone deficiency, its administration may have a therapeutic role in LUTS treatment [**6**][**7**].

At the same time, there are still gaps that make it challenging to perform comparative studies, starting with the standardization of terminology, different methods of assessing the clinical situations and, in particular, different treatment concepts [**8**].

Therefore, the association between LUTS and erectile dysfunction is a problem of contemporary urology with many unknown uncertainties.

**Purpose of the study:** To assess the prevalence of lower urinary symptoms and the possible correlations between it and erectile dysfunction in men from the Republic of Moldova.

## Material and methods

A transversal descriptive study was conducted between 2015 and 2016, on the basis of representative sample questionnaire of 1,186 males aged between 18-80 years. The mean age of the subjects enrolled in the study was 47.8 years. LUTS symptomatology presence was determined by completing the answers to questions that characterize micturition, as well as validated IPSS questionnaires, which determine the presence and severity of low urinary complaints specific to the presence of benign prostatic hypertrophy, prostate cancer or inflammatory diseases of the prostate and urethra. The presence of erectile dysfunction was confirmed using the IIEF-5 (International Index of Erectile Function) questionnaire.

The study design was approved by the Research Ethics Committee of the SUMPh "Nicolae Testemitanu", Chisinau, Republic of Moldova.

The data were processed using the statistical software package SPSS 21. The descriptive, comparative (Student t-test) and Pearson’s R correlation coefficient statistics were used. The significant threshold for comparisons was set at 5% (p <0.05).

## Results

From 1,186 investigated men, 626 (52.8%) reported the complete absence of low urinary complaints, but 560 (47.2%) had at least one complaint. The most common reported urinary complaint was nocturia, present in about 41% of men, followed by the need to force urination, 20.4% and frequent urination in less than 2 hours from the last urination in 18.5%, 190 people (16%) reported the incomplete discharge of bladder or residual urine, characteristic to the presence of infra bladder obstruction. At the same time, 83 (7%) respondents reported the presence of being a characteristic to an inflammatory process. The prevalence of lower urinary tract symptoms is statistically different in subjects up to 40 years of age, where about 122 patients (23.4%) reported the occurrence of some complaints, especially irritating ones, and over this age, when about 438 people, 65.9% people reported the presence of urinary complaints, including obstructive ones (p <0.05). The prevalence of some LUTS symptoms is presented in **Table 1**.

**Table 1:** The presence of complaints characteristic to low urinary symptomatology (1,186 respondents)

	Under 40 years		Over 40 years		Total	
Age						
Complaint	n	%	n	%	n	%
Incomplete emptying feeling	29	5.6%	161	24%	190	16%
Frequent urination: less than 2 hours since the last urination	38	7.3%	181	27.2 %	219	18.5%
Difficulty to postpone urination or urine loss	6	1.2%	76	11.4%	82	6.9%
Urinary intermittency	12	2.4%	78	11.7%	90	7.6%
Weak urinary jet	21	4 %	196	29.5 %	217	18.3%
Necessity to force urination	27	5.2 %	215	32.3%	242	20.4 %
Nocturia	76	14.6%	412	62%	488	41.1%
Testicular, penile, perineal pain.	26	5%	48	7.2%	74	6.2%
Burning sensation with urination	27	5.2%	48	7.2%	75	6.3%
Any symptom ofthose enumerated	262	0.5 complaints per male	1.415	2.2 complaints per male	1.677	1.4 complaints per male

A lot of respondents reported the existence of many overlapping complaints, but as it is shown in the table, the average number of complaints per respondent can be 0.5 for a man under 40 and about 2.2 for a man over this age.

For a more objective assessment of low urinary complaints, the benchmark is the IPSS score. The IPSS score is primarily specific to prostate pathologies and is based on summarizing the patient's answers to 7 questions that characterize urination, but the 8th question refers to the quality of life. Questions describe bladder emptying, urinary frequency, urinary excretion and urine flow, etc. Each question has several response choices with a definite score, following the calculation of the score, as recommended by the American Urology Association (AUA), prostate symptomatology classifies as mild, medium and severe. The mild symptomatology corresponds to an IPSS score of up to 7, but the quality of life, QoL index up to 2. For the mean symptomatology, the IPSS index is greater than 7 but less than 19, and for a severe symptomatology, the IPSS is greater than 20, QoL for both being greater than 2.

The IPSS score has a higher degree of accuracy in the urological patient's assessment and is therefore both clinically and scientifically relevant. Typically, in the case of mild IPSS, the therapy is under the family doctor's observation, but medium and severe IPSS compulsorily implies more detailed urological assessment.

Following the assessment of responses, the IPSS score was present in men aged 40 years and older in 82 participants (15.8%), most of them with slight, significantly lower degree compared to those aged 40 years and over, where 416 (62.6%) persons experienced IPSS symptomatology, and over 65, about 86% of people had IPSS complaints (**Table 2**).

**Table 2:** The presence of low urinary complaints according to the IPSS score

Age (years)	IPSS mild		IPSS medium		IPSS severe		Total	
	n	%	n	%	n	%	n	%
18 – 29	15	5.8%	5	2%	0	-	20	7.8%
30 – 39	38	15.6%	14	5.4%	10	3.8%	62	24%
40 – 49	64	23.6%	23	8.8%	17	6.5%	104	38.7%
50 – 64	118	41%	57	19.9%	45	15.8%	220	76%
65	24	22.4%	37	34.6%	31	30%	92	86%
Total	259	21.8%	136	11.7%	103	8.9%	498	42.9%

The difference between the number of men who were evaluated according to IPSS criterion and those who had low urinary complaints is explained by the fact that the complaints characteristic to chronic prostatitis such as pain or urination burning sensation are part of chronic prostatitis index NIH CPSI.

Erectile function was evaluated according to the IIEF-5 questionnaire validated to assess the prevalence of the disease. According to the international index of erectile function, the total prevalence of erectile dysfunction was reported in 47.1% of the population, with a difference between the age groups, 17.3% in patients up to 30 years and 88.8% in ones over 65 years old (P <0.001) (**Table 3**). Also, the prevalence of severe erectile dysfunction is significantly higher in older men, 30.8% in those over 65, compared to 0.7% in those aged up to 30 years (P <0.001).

**Table 3:** Prevalence and manifestation degree of erectile dysfunction in age groups

ED presence and degree	Age (years)					Total
	18-29	30-39	40-49	50-64	>65	
Severe	2 (0.7%)	3 (1.2%)	6(2.2%)	17 (5.9%)	33 (30.8%)	61 (5.1%)
Moderate	4 (1.4%)	7(2.7%)	15 (5.5%)	45 (15.7%)	19 (17.8%)	90 (7.6%)
Slightly moderate	8 (3.0%)	15 (5.8%)	38 (14.0%)	63 (22%)	23 (21.5%)	147 (12.4%)
Mild	31 (11.9%)	45 (17.3%)	82 (30.2%)	83 (28.9 %)	20 (18.7%)	261 (22%)
Without erectile dysfunction	216 (82.7%)	190 (73%)	130 (48%)	79(27.5%)	12 (11.2%)	627 (52.9%)
Total	261	260	271	287	107	1.186

Erectile dysfunction prevalence in men with few urinary complaints was analyzed.

In the first stage, we evaluated the presence of erectile dysfunction in patients with nocturnal pollakiuria. Nocturnal pollakiuria is one of the most significant symptoms characteristic of a urinary tract disorder, but also neurological or cardiovascular disorders. Normally, it is absent or accepted once, but only at certain times and conditions. As mentioned previously, 698 men or about 59% reported the absence of night-time urination. In this group, erectile dysfunction was present in 32.7% of people. At the other end, there are 201 people who reported 2, 3 or more night-time urinations. In this group the prevalence of erectile dysfunction was 86% (P<0.001) (**Table 4**).

**Table 4:** Prevalence and severity degree of ED in men who reported nocturnal pollakiuria

Symptoms	Without nocturnal urination		Nocturnal urination once		Nocturnal urination twice		3 and more nocturnal urinations		Total	
	**n**	**%**	**n**	**%**	**n**	**%**	**n**	**%**	**n**	**%**
Severe ED	9	1.1%	16	5.6 %	18	11.8%	18	37,5%	61	5,2%
Moderate ED	21	2.4%	23	8 %	37	24.1%	9	18,8%	90	7,6%
Easily moderate ED	50	7.2%	49	17.1%	41	26.8%	7	14,6%	147	12,4%
Mild ED	145	20.8%	72	24.9%	33	21.5%	10	20,8%	261	23%
Without ED	473	67.3%	127	44.3%	24	17.9%	4	8,3%	627	52,9%
Total	698		287		153		48		1.186	

There is a direct dependence between presence and degree of manifestation of nocturnal pollakiuria and erectile dysfunction. Of the patients who have no night urination, only about 1% will have severe erectile dysfunction, compared to 37.5% of those with nocturnal pollakiuria, and only 8.3% of patients will have no erectile dysfunction at all.

At the same time, nocturnal pollakiuria is only a symptom of LUTS, which does not fully reflect the severity of the disease, the IPSS score being much more relevant. Therefore, the prevalence and extent of erectile dysfunction in correlation with the IPSS score were determined. In 688 patients reporting the absence of urinary symptomatology (with negative IPSS), erectile dysfunction was present in 177 subjects (25.7%), among them in mild form. From 498 men with a current IPSS score, only 23.3% did not have erectile dysfunction (p <0.05). The presence and severity of erectile dysfunction depend on the severity of IPSS. In 239 subjects with an IPSS score higher than 8, erectile dysfunction was present in 207 (86.6%), of which only in 23.2% a mild form, but in patients with severe IPSS, only 2.9% did not have erectile dysfunction, compared with 32.4% in those with mild IPSS, or 74.3% (p <0.05) who did not generally report urinary symptomatology (**Table 5**).

**Table 5:** Prevalence and severity degree of erectile dysfunction according to the IPSS score

IIEF. n(%)						
IPSS	Severe	Moderate	Slightly moderate	Mild	Without ED	Total
Without IPSS	3 (0.43%)	10 (1.5%)	35 (5.1%)	129 (18.8%)	511 (74.3%)	688
IPSS present	58 (11. 6%)	80 (16.1%)	112 (22.5 %)	132 (26.5%)	116 (23.3%)	498
Mild	14 (5.4%)	24 (9.3%)	53 (20.5%)	84 (32.4%)	84 (32.4%)	259
Moderate	17 (12.5%)	25 (18.4%)	38 (28%)	27 (19.9%)	29 (21.3%)	136
Severe	27 (26.2%)	31 (30.1%)	21 (20.4%)	21 (20.4%)	3 (2.9%)	103
Total	61 (5.1%)	90 (7.6%)	147 (12.4%)	261 (22%)	627 (52.9%)	1.186

We also analyzed the presence of erectile dysfunction in patients with age-related LUTS symptomatology. To simplify the interpretation of the results, we divided the respondents into two groups, up to 40 years old and older than 40 (**Table 6**). Thus, in patients up to 40 years old, IPSS symptomatology was present in 82 males (15.2%) and absent in 439 (84.8%). There were no signs of erectile dysfunction in men with IPSS of 41 (45%), and in those with ED - 9 men (10.9%) reported moderate or severe dysfunction that is significantly higher compared to those without IPSS, 1.6%, but much lower than in people over 40. Thus, 416 (62.6%) of people over the age of 40, who reported an IPSS score presence, only 18% did not report ED. So at a young age, in the absence of other risk factors, only the presence of IPSS correlates less with ED compared with elderly men.

**Table 6:** Prevalence and severity degree of erectile dysfunction depending on IPSS presence and age

Age	IPSS				IIFE – 5			Total
			severe	moderate	Slightly moderate	Mild	Without ED	
Under 40	Absent IPSS	n	2	5	9	58	365	439
		%	0.5%	1.1%	2.1%	13.2%	83.1 %	84,2%
	Present IPSS	n	3	6	14	18	41	82
		%	3.6%	7.3%	17.1%	22%	50%	8%
	Total	n	5	11	23	76	406	521
		%	1%	2.1%	4.4%	14.6%	77.9%	
Over 40	IPSS Absent	n	1	5	26	71	146	249
		%	0.4%	2%	10.4%	28.5%	58.6%	37,4%
	IPSS Present	n	55	74	98	114	75	416
		%	13.2%	17.8%	23.6%	27.4%	18%	62,6 %
	Total	n	56	79	124	185	221	665
		%	8.4%	11.9%	18.3%	26.5 %	33.4%	
Total			61	90	147	261	627	1.186

The severity of IPSS in elderly men will more evidently influence erectile dysfunction. Thus, severe or moderate ED will have about 30.1% of men with present IPSS, compared with 2.4% men whose urinary symptomatology is absent.

The presence of an IPSS score in young people is mostly owed to urethritis or prostatitis and does not directly contribute, at least during initial stages of disease manifestation, to the occurrence of an erectile dysfunction, especially if it is of an easy or moderate degree.

Correlations between IPSS severity and ED degree were also studied and presented in **Table 7**.

**Table 7:** Prevalence and degree of IPSS and erectile dysfunction depending on age

				IIFE – 5			
Age	IPSS						Total
		Severe	Moderate	Slightly moderate	Mild	Without ED	
Under 40	Without IPSS	2 (0.5%)	5 (1.1 %)	9 (2.1%)	58 (13.2%)	365 (83.1%)	439 (84.2%)
	Slight	0	3 (5.7%)	7 (13.2%)	11 (20.8%)	32 (60.4%)	53
	Medium	1 (5.3%)	2 (10.5%)	3 (15.8%)	5 (26.3%)	8 (42.1%)	19
	Severe	2 (20%)	1 (10%)	4 (40%)	2 (20%)	1 (10%)	10
	Total IPSS	3 (3.6%)	6 (7.3%)	14 (17.1%)	18 (18.3%)	41 (50 %)	82 (15.8%)
	Total	5 (1%)	11 (2.1%)	23 (4.4%)	76 (14.6%)	406 (77.9%)	521 (100%)
Over 40	Without IPSS	1 (0.4%)	5 (2%)	26 (10.4%)	71 (28.5%)	146 (58.6%)	249 (37.4%)
	Slight	14 (6.8%)	21(10.2%)	46 (22.3%)	73 (35.4%)	52 (25.2%)	206
	Medium	16(13.7%)	23 (19.7%)	35 (31.6%)	22 (18.9%)	21 (17.9%)	117
	Severe	25 (26.9%)	30 (32.3%)	17 (18.3%)	19 (20.4%)	2 (2.2%)	93
	Total IPSS	55 (13.2%)	74 (17.8%)	98 (23.6%)	114 (27.4%)	75 (18%)	416 (62.6%)
	Total	56 (8.4%)	79 (11.9%)	124 (18.6%)	185 (27.8%)	221 (33.2%)	665
Total		61	90	147	261	627	1.186

It is clear from the table that no matter the patient’s age, the IPSS severity directly influences the severity of erectile dysfunction, but more obviously in those over 40. Thus, 20% of people up to 40 years old and 26.9% of those over 40 with severe IPSS will also have severe erectile dysfunction. At the same time, mild IPSS does not correlate with severe ED in the young, only 5.7% reporting a severe or moderate erectile dysfunction, compared to 17% of those over 40 years old.

Correlations between IIEF -5 variables score and IPSS total, according to the Pearson coefficient, showed an inversely proportional relationship (r = - 0.575, P 0.0001) between these parameters. An increased IPSS score leads to an IIEF-5 score decrease. Therefore, patients with advanced LUTS symptomatology manifested through IPSS increase will also have a more pronounced erectile dysfunction. The statistic significance is more evident in patients over 40 (**Table 8**).

**Table 8:** Correlations between erectile dysfunction and IPSS score (1,186 patients)

Age (years)	No. patients	Mean ± Standard Deviation		Correlation IPSS and IIFE-5	
		IPSS	IIFE-5	R	P
18 – 39	521	3.65± 5.36	20.60±5.28	- 0.153	P < 0.05
40 – 49	271	8.7 ± 9.52	16.81±5.46	- 0.587	P < 0.001
50 – 64	287	12.47±8.49	13.9±5.84	- 0.481	P < 0.001
64 +	107	18.9 ± 8.31	6.92±3.41	- 0.210	P < 0.001
Total	1186	13.25± 9.29	13.8 ±6.36	- 0.575	P < 0.001

## Discussions

As it was mentioned in the introduction of this paper, LUTS prevalence studies, with or without correlations with erectile dysfunction, were performed in several countries. These publications appreciate the dependence of erectile dysfunction on LUTS symptomatology severity. At the same time, the data vary greatly from one country to another, probably depending on the socio-economic situation of the country. Thus, a Nigerian study realized on a sample of 658 men over 40 years of age determined only LUTS global prevalence, which was 59.1%, with 48.2% predominance of bladder filling symptoms comparative with other LUTS signs. Only half of the men suffering from LUTS searched for medical assistance, the latter being dependent on educational status [**9**].

In Gonsalez et al.’s study, conducted in Mexico on a sample of 1,041 men with an average age of 48.6 ± 14.5, only 11.8% men were asymptomatic, but 7.8% had a severe IPSS score, nocturia being present in 72.4% of people. At the same time, 63.2% people also reported the presence of erectile dysfunction [**10**].

The prevalence of moderate and severe LUTS in men from Hong Kong being under primary care was 36% and 36% respectively, and erectile dysfunction was found in 68%, including 28% with a severe form. We mention that it concerns the men who already suffered from a certain pathology, compared to our study, which is a population survey [**11**].

The population survey conducted in the Russian Federation showed the presence of LUTS symptomatology according to the IPSS score in 59.9% of males over 20 and the prevalence of erectile dysfunction of 48.9%. The LUTS symptomatology was more commonly associated with erectile dysfunction than high blood pressure or diabetes mellitus. [**12**] Song’s study, based on a sample of 1,644 men aged over 50, showed obvious direct correlations between LUTS and ED severity, so men with severe IPSS will and even slight IPSS will contribute to dysfunction appearance in about 85.7% [**13**.]

At the same time, the presence of erectile dysfunction can be a predictor of LUTS severity. Patients with severe LUTS often suffer from other pathologies, which are considered risk factors for erectile dysfunction, such as obesity, high blood pressure or metabolic syndrome [**14**].

The statistically significant correlations between LUTS and ED require comprehensive primary patient assessment for one of the above-mentioned pathologies including the use of IPSS and IIFE standardized questionnaires as well as complex diagnosis methods for simultaneous diagnosis of both. Taking into consideration that LUTS treatment can affect sexual function, sexual appetite, ejaculation and erection, primary examination of the patient is mandatory. On the other hand, [**15**] LUTS symptomatology presence has a significant impact on the quality of life, including the sexual one. On the other hand, most elderly patients consider their presence a part of the aging process and avoid addressing a doctor.

LUTS symptomatology may occur due to adenoma or prostate adenocarcinoma, or chronic prostatitis, requiring long-term treatment, sometimes altering erectile function. On the other hand, treatment can improve LUTS symptomatology and later the sexual function.

The sexual function should be evaluated in all men requiring LUTS treatment using validated questionnaires. It is also necessary to assess the presence of comorbidities and used medication, lifestyle and other risk factors [**16**][**17**].

## Conclusions

LUTS and ED symptomatology have an increased prevalence among men in the Republic of Moldova, especially after the age of 40. Statistically significant correlations were demonstrated between these two pathologies. These correlations need to be taken into account both in establishing the diagnosis and in concomitant treatment.

ED total prevalence in the studied population was 47.1%, including mild - 22%, slightly-moderate - 12.4%, moderate - 7.6% and severe - 5.1%.

The overall prevalence of LUTS symptomatology, according to the IPSS score, was 42.9%, with significant variations in males up to 40 years old - 15.7% and over this age - 62.6% (p <0.05). LUTS presence correlates directly with erectile dysfunction prevalence, especially in men over the age of 40, where 82% of those with LUTS will also have ED.
